# Natural Fibres and Their Composites

**DOI:** 10.3390/polym12102380

**Published:** 2020-10-15

**Authors:** Vincenzo Fiore

**Affiliations:** Department of Engineering, University of Palermo, Viale delle Scienze, Edificio 6, 90128 Palermo, Italy; vincenzo.fiore@unipa.it

Due to several promising properties, such as their low density and specific properties, low price, easy processing, health advantages, renewability and recyclability, increasing attention was paid in the last years to natural fibres as alternatives to synthetic counterparts for the reinforcement of polymeric based composites.

On the other hand, it is well known that the hydrophilic nature of natural fibres leads to high susceptibility to moisture absorption and low resistance to humid and wet environmental conditions. Moreover, these fibres evidence limited and highly variable mechanical properties as well as weak adhesion with hydrophobic polymers. For these reasons, the use of natural fibres in industrial applications such as automotive, marine and infrastructure, are often limited to non-structural or semi-structural interior components. 

To overcome these drawbacks, natural fibres can be pre-tread or used together with specific additive and/or synthetic fibres. These approaches have been widely exploited in literature, and the resulting composites have shown a suitable balance of mechanical properties, thermal stability, ageing tolerance against humid or aggressive environments, cost and environment care. 

In this context, the present Special Issue comprises fourteen peer-reviewed original research articles about polymer composite materials reinforced with natural fibres. 

The main topic includes the investigation of the effect of novel lignocellulosic reinforcements and nano or micro additives on performances of thermoplastic or thermoset based composites. 

The use of novel lignocellulosic fibres is studied in several contributions to this Special Issue. The team of Andrea Zille [1] analyzes the effect of adding dog wool fibres on the properties of polyurethane (PU)-based eco-composite foam. They show that tensile and compression strengths, hydration capacity and thermal capacity are improved whereas the foam dilatation with heating decreased with increasing the amount of dog wool microparticles, thus demonstrating the potential of this animal-derived waste for insulation applications, with a low cost and minimal environmental impact. Mina Hernandez et al. [2] develop a fully bio-based composite using a natural resin obtained from Mopa-Mopa (Elaeagia Pastoensis Mora) plant as matrix and fique fibres as reinforcement. Thanks to easy processing and good physicochemical and mechanical characteristics, it is shown that the bio-based composite can be used as wood–plastic for the replacement of plastic and/or natural wood products widely used today in several applications. Similarly, Pompei et al. [3] show that the introduction of leather fragments in a self-produced thermoplastic starch (TPS) based on starch plasticized with glycerol and cross-linked using citric acid proved to be promising. The composite biodegradability allows its possible application in products where contact with soil and progressive non-toxic degradation is required, such as it is the case for the on-site production of mulching films.

Wang et al. [4] studied the relationships between the working fluids, process characteristics and products from the modified coaxial electrospinning of zein focusing their attention on the control of the processing process during the manufacturing process as well as on the prediction and maintenance of the nanofibre quality. In particular, using an electrospinnable zein solution as the core fluid and LiCl solutions as the sheath working liquids, a series of modified coaxial electrospinning processes are performed, thus successfully preparing a number of zein nanoribbons. In a further paper [5] concerning the use of novel lignocellulosic fibres, a mixture of thermoplastic polybutylene succinate (PBS), tapioca starch, glycerol and empty fruit bunch fibre was prepared by a melt compounding method and characterized by means of mechanical, thermal and immersion tests.

The effect of the addition of nano or micro additive as filler in natural fibre reinforced composite is investigated in several papers [6,7,8,9]. 

In this context, the team of Andrea Lazzeri [6] prepared plasticized poly(lactic acid) (PLA)/poly(butylene succinate) (PBS) blend-based films containing chitin nanofibrils and calcium carbonate by extrusion and compression moulding methods. The diffusion coefficient experimental data shows that the adding of chitin nanofibrils can slow the plasticizer migration. However, the best result was achieved with micrometric calcium carbonate while nanometric calcium carbonate results less effective due to bio polyesters’ chain scission. On the other hand, the use of both micrometric calcium carbonate and chitin nanofibrils was counterproductive due to the agglomeration phenomena that were observed. Russo et al. [7] evaluate the effects of two carbon nanostructures (graphene nanoplatelets (GNPs) and carbon nanotubes (CNTs)), of a chemical modification with a fatty acid and of maleated polypropylene, with the aim of mitigating the highly hydrophilic nature of flax fibres thus increasing their compatibility with apolar polypropylene. On the bases of the experimental data, the authors state that these simple treatments, potentially prone to further optimization, can represent a step toward producing natural fibre composites with mechanical profiles compatible with semi-structural applications. Similarly, Wang et al. [8] improved the hygrothermal resistance of flax fibre reinforced epoxy composites through grafting flax fabric with nanoclay. In more detail, the introduction of nano-clay reduces both moisture uptake and the coefficient of diffusion of composites that show better dimensional stability than the untreated ones.

In the paper by Costa et al. [9], the performance of graphene oxide coated curaua fibre reinforced epoxy composites in a multilayered armour system intended for ballistic protection is evaluated showing that this innovative composite attends the standard ballistic requirement remaining intact, differently from the non-coated curaua fibre similar composite.

Another topic addressed in this special issue deals with the use of natural fibres in hybrid composites useful for different applications. Dhakal et al. [10] present an interesting study on the evaluation of the low-velocity falling weight impact behaviour of flax-basalt vinyl ester (VE) hybrid composites, showing that the hybrid system possesses higher impact energy and peak load than flax fibre reinforced composite. Hence, the experimental results indicate that the hybridization is a promising strategy for enhancing the toughness properties of natural fibre composites. Flax, Basalt, E-Glass FRP and their hybrid combinations are used to strengthen wood beams in the paper by Wang et al. [11]. The bending tests performed on strengthened wood beams shows that all hybrid FRPs exhibit no significant enhancement in load carrying capacity but larger maximum deflection compared to the single type of FRP composite.

The last three papers belonging to this special issue deals with the investigation of flexural properties and impact damage behaviour of basalt fibre reinforced polypropylene composites [12], the mechanical and rheological behaviour of composites reinforced with different natural fibres [13] and the theoretical modelling of the stiffness of recycled cotton fibres reinforced polypropylene composites [14]. In particular, the paper by Serra et al. [14] highlight the opportunity of recovering textile cotton fibres, useless for the textile industry, to obtain composite materials with promising performances. Moreover, the use of two different micromechanics models allowed evaluating the impact of the morphology of the fibres on Young’s modulus of a composite.

In summary, the papers contributed to this Special Issue give some very nice insights on the use of natural fibres as reinforcement of composite materials thus making this volume useful for students as well as for designers and engineers that would like to develop a deeper understanding on the use of this important and emerging research subject.
